# Belladonna in Aphonia Cases

**Published:** 1874-03

**Authors:** T. Curtis Smith

**Affiliations:** Middleport, Ohio


### BELLADONNA IN APHONIA CASES.

BY T. CURTIS SMITH, M.D., MIDDLEPORT, OHIO.

In February, 1873, I was called to see Mrs. E., who had recently been afflicted with measles. She was æt twenty-eight years, mother of two children, and usually enjoyed very good health in every respect. Temperament nervous. In connection with and for a fortnight subsequent to the attack of rubeola, she had also an irritative fever of a rather asthenic grade. During all this time and for four weeks subsequent to the subsidence of the fever, she was attended by a "socalled doctor" of the *infinitesimal* persuasion. At the time of my first visit she had not been able to speak above a whisper, and that with much effort, for five or six weeks. Examination showed much general debility, and also the mucus membrane of the pharynx, as far as could be seen, greatly congested and apparently thickened. From this fact, and the existence of aphonia, I believed the mucous larynx was also greatly hyperemic, I placed her on general tonics, and directed the fl. ext. belladonna to be taken in small and often repeated doses till the throat and mouth should become very dry. On the second day after this, I called, finding her pretty fully atropinized and able to speak audibly, but with some effort. The voice rapidly improved however, and in a week she could talk with quite as much ease as ever. Her general strength however, was only returning slowly and she was still pale and weak, so that it could not have been the general tonics that restored the voice so soon.

A few days later I was called to Clifton, W. Va., to see a boy æt. four years, who had but just recovered from measles, but the sequence of aphonia still clung to him, I placed him on the same agent as the first case. In two days he ceased to whisper and began to speak audibly, his voice in a few hours thereafter becoming quite normal. The agent was continued but forty-eight hours.

In March of the same year, I saw a third case in a married lady who, for ten days subsequent to the complete subsidence of all symptoms of rubeola, was still afflicted with aphonia. Her general health was not then greatly below par, so I simply gave the belladonna alone. As soon as the throat became thoroughly affected with the sensation of dryness, the voice became audible and was soon fully restored.

I give these cases without note or comment. I was led to use the agent from its known influence over the mucous lining of the throat and mouth, and from having observed its beneficial effects on the same in a hyperemic and inflamed condition.

